# Understanding women’s motivations to participate in MTN-003/VOICE, a phase 2b HIV prevention trial with low adherence

**DOI:** 10.1186/s12905-019-0713-6

**Published:** 2019-01-25

**Authors:** Ariana W. K. Katz, Barbara S. Mensch, Kubashni Woeber, Petina Musara, Juliane Etima, Ariane van der Straten

**Affiliations:** 1 Women’s Global Health Imperative, RTI International, 351 California St, Suite 500, San Francisco, CA 94104 USA; 2 Population Council, New York, NY USA; 3 South African Medical Research Council, HIV Prevention Research Unit, Durban, South Africa; 4 University of Zimbabwe College of Health Sciences Clinical Trials Research Centre (UZCHS-CTRC), Harare, Zimbabwe; 5 Makerere University-Johns Hopkins University Research Collaboration, Kampala, Uganda; 6 Center for AIDS Prevention Studies (CAPS), University of California San Francisco, San Francisco, CA USA

**Keywords:** Motivation to participate, HIV prevention, Clinical trials, VOICE trial, Microbicides, Oral PrEP

## Abstract

**Background:**

In biomedical prevention trials, correct and consistent use of the investigational product is crucial to determine efficacy. Product adherence in VOICE, a phase 2B randomized trial of a vaginal gel and oral tablets for HIV prevention, was low (~ 34%), yet self-reported adherence and retention was high (> 90%). This analysis from VOICE-D, a post-trial qualitative ancillary study, explores motivations to participate in VOICE, and possible sources of misalignment between the stated priorities of the trial and the participants.

**Methods:**

VOICE-D enrolled 171 former VOICE participants to investigate, among other things, reasons for joining and remaining in the trial. Local language in-depth interviews and focus groups were transcribed and translated into English and coded and analyzed using NVivo. Data on motivation to join obtained from a VOICE termination visit survey of 106 participants were also analyzed to corroborate the VOICE-D findings.

**Results:**

Participants primarily participated for personal health benefits (e.g. free healthcare and HIV testing) and reported remaining enrolled from a sense of commitment to the trial. Altruistic motivations were the most commonly stated motivation on the termination visit survey; qualitatively, many of those stating altruistic reasons also desired personal health benefits. Joining for financial reimbursement was not commonly mentioned. Social networks influenced recruitment and spread therapeutic misconception.

**Conclusions:**

Women’s participation for personal health benefits highlighted their desire to monitor their HIV risk and overall health. Helping participants view use of investigational products as improving social capital and reminding participants of their study responsibilities may improve trial outcomes. Understanding the reasons for participating in studies will help to ensure alignment between priorities of researchers and participants.

**Trial registration:**

NCT02358616; Posted February 9, 2015, retrospectively registered.

## Background

HIV prevention trials rely on healthy volunteers to investigate new interventions designed to decrease risk of viral acquisition. Joining and remaining in clinical trials, however, does not solely fulfill the participants’ obligations to the research. Indeed, in biomedical prevention trials, correct and consistent use of the investigational product is also crucial to determine efficacy [1–4]. While the primary objective of clinical trialists is to determine if a new product or intervention is safe and efficacious, the priorities of the participants can be multifaceted.

Pre-Exposure Prophylaxis (PrEP) can be highly effective for HIV prevention [5–8], including for women [9], yet results from several tenofovir-based PrEP trials in female populations in sub-Saharan Africa have been disappointing, driven by low adherence to product use [10–12]. The VOICE trial was a phase 2B five-arm placebo-controlled randomized trial, which investigated the effectiveness of daily use of antiretroviral oral tablets or daily use of vaginal tenofovir gel for preventing male-to-female sexual transmission of HIV-1. Results from VOICE found none of the drug regimens effective in reducing HIV-1 acquisition in the intent-to-treat analysis. Further analysis revealed that product adherence was low (estimated at only 34%), despite high self-reported adherence and overall trial retention (> 90%), and low amounts of returned unused products [10]. To further explore these results, a qualitative ancillary study, MTN-003D /VOICE-D, was implemented after the completion of VOICE. VOICE-D investigated, among other things, reasons for joining the trial and for non-adherence to product use during VOICE. Details from participants reports indicated a range of product (non)adherence patterns, including noninitiation, discontinuation, and misimplementation of daily dosing (e.g. modified dosing or regimen, variable use and visit-driven use) corroborating biomarker data suggestive of inconsistent low level product use [13, 14]. Reasons for misreporting adherence included human nature, not wanting to look like a “bad” participant to study staff, fear of negative consequences, ease of getting away with misreporting, and avoiding inconvenient additional counseling [15]. Participants were more forthcoming about adherence challenges in Stage 2 following receipt of objective drug test results [16]. This paper uses the VOICE-D sample to focus on motivations to join and remain in the VOICE trial, and possible sources of misalignment between the stated priorities of participants and those of the trial.

## Methods

### MTN-003/VOICE: Termination visit vaginal or oral product behavioral questionnaire (TVB/TOB) data on motivation to join

VOICE was conducted at 15 sites with 5029 participants in South Africa, Uganda, and Zimbabwe between 2009 and 2012. In the VOICE trial, the oral Tenofovir disoproxil and vaginal Tenofovir gel arms were stopped early due to futility. Further details of the trial design, procedures, and primary findings were previously published [10]. From December 2011 to August 2012, during the last 9 months of the trial, a **T**ermination visit **V**aginal or **O**ral product **B**ehavioral questionnaire (TVB/TOB) was implemented, following early closure of the oral tenofovir and vaginal gel arms [17, 18]. The TVB/TOB questionnaire was administered to 2321 participants (46%) who were still being followed in the VOICE trial [19], and included the following question: “Why did you participate in the VOICE trial?” Six categorical response options were provided, and multiple responses were permitted (see Table 2).

### MTN-003D/VOICE-D: Participants and procedures

VOICE-D, a 2-stage qualitative sub-study, was designed after the early closure of the oral and vaginal tenofovir arms in VOICE to explore factors that may have contributed to the dilution of efficacy. One hundred seventy-one former VOICE participants were enrolled 18–33 months after VOICE, between December 2012 and March 2014. VOICE-D took place at four sites in all three VOICE countries, including Kampala, Uganda (*n* = 61); Durban, South Africa (*n* = 45); and Harare, Zimbabwe (*n* = 65) (see Table 1).

**Table 1:** Demographic information of VOICE-D participants in either stage (*n* = 171)

	Total (*n* = 171)	Durban, South Africa (*n* = 47, 27%)	Harare, Zimbabwe (*n* = 65, 38%)	Kampala, Uganda (*n* = 59, 35%)	Difference by Country^Ϯ^
Age					
Median (mean, min-max)	28 (28.9, 20–41)	25 (26.8, 20–40)	29 (29.6, 21–41)	29 (29.9, 20–41)	**0.0008**
Married					
Yes	92 (54%)	0 (0%)	57 (88%)	35 (59%)	**< 0.0001**
No	79 (46%)	47 (100%)	8 (12%)	24 (41%)	
If unmarried (*n* = 79), has primary sex partner	68 (86%)	43 (91%)	6 (75%)	19 (79%)	0.2147
Partner has other sexual partners					
Yes	48 (30%)	6 (14%)	12 (19%)	30 (56%)	**< 0.0001**
No	12 (8%)	3 (7%)	3 (5%)	6 (11%)	
Don’t know	100 (63%)	34 (79%)	48 (76%)	18 (33%)	
# of lifetime sex partners^b^					
Median (mean, min-max)	3 (10.8, 1–99+)	2 (3.4, 1–15)	1 (2.4, 1–35)	5 (26.1, 2–99+)	**< 0.0001**
# of children alive at birth					
Median (mean, min-max)	2 (2.1, 0–6)	1 (1.3, 0–4)	2 (2.3, 1–5)	2 (2.5, 0–6)	**< 0.0001**
# of children taking care of					
Median (mean, min-max)	2 (2.3, 0–6)	1 (1.5, 0–6)	3 (2.7, 1–6)	3 (2.7, 1–5)	**< 0.0001**
Completed secondary school or more	68 (40%)	20 (43%)	28 (43%)	55 (93%)	**< 0.0001**
Earns own income	125 (73%)	40 (85%)	35 (54%)	50 (85%)	**< 0.0001**
Method of earning income^a^					
Formal employment	28 (22%)	9 (23%)	6 (17%)	13 (26%)	0.6616
Self-employment	67 (54%)	1 (3%)	29 (83%)	37 (74%)	**< 0.0001**
Other	33 (26%)	33 (83%)	0 (0%)	0 (0%)	**< 0.0001**
Partner provides financial/material support	146 (91%)	36 (84%)	58 (92%)	52 (96%)	0.1036
SES Score^c^					
Lowest 40%	68 (40%)	7 (15%)	16 (25%)	45 (76%)	**< 0.0001**
Middle	70 (41%)	18 (38%)	38 (58%)	14 (24%)	
Highest 20%	33 (19%)	22 (47%)	11 (17%)	0 (0%)	

^a^Participants with 99 or more sex partners were recorded as ‘99’; ^b^Multiple answers allowed; ^c^An SES indicator variable was created using principal component analysis (PCA) of 10 demographic assets from the VOICE-D CRF including: home ownership; number of rooms in household; household assets of electricity, radio, television, mobile telephone, non-mobile telephone, refrigerator; toilet facilities; and drinking water sources [50]. A tri-level categorical variable (lowest 40%, middle 40%, and highest 20%) was created based on the first eigenvalue and the SAS-generated PRIN1 score [51]; ^Ϯ^*P*-value from Fisher’s Exact test for categorical variables and Kruskal-Wallis test for continuous variables.

Bold text signifies p<0.05.

Stage 1 included single in-depth interviews (IDI) with pre-selected former VOICE participants (*n* = 88) to explore adherence to study products, and potentially sensitive behaviors, including anal sex [20–22]. To recruit the former VOICE participants, the VOICE Data Coordinating Center generated randomly selected lists, stratified by participant characteristics such as anal sex behavior and HIV status, for those who had provided permission to be contacted and had been on product for a minimum of 3 months during VOICE. Site staff contacted individuals sequentially on the lists until target numbers (20 per site) were met. Stage 2 was added once final VOICE results had been released and participants’ individual drug pharmacokinetic results were available [14–16], during which participants completed either an IDI only (*n* = 55), participated in a focus group discussion (FGD) only (*n* = 59) or took part in both (*n* = 13). Similar to Stage 1, participants for Stage 2 were recruited from a randomly selected list, generated from the Data Coordinating Center for those who had provided permission to be contacted and, unique to Stage 2, had available pharmacokinetic data. The lists were stratified by HIV status, study group, and drug pharmacokinetic detection levels [14]. Site staff contacted potential participants in ascending order of the lists until target numbers (36–48 per site) were met. The target sample sizes were based on ensuring adequate participant representation in each study group (tablets and gel), and within other stratification criteria for each stage (i.e., risk behavior, HIV status, drug detection levels) and based on practical/feasibility considerations.

A total of 12 FGDs occurred, 4 at each site, comprised of 4 to 10 participants per FGD. To further explore product experiences and adherence challenges, trained female research staff privately presented participants with their pharmacokinetic results (either during the IDI or before the FGD). Demographic characteristics for this VOICE-D sample were drawn from a questionnaire administered prior to the qualitative interview. IDIs and FGDs were conducted in the participant’s language (Luganda, Shona, Zulu, or English) by trained qualitative interviewers and facilitators following semi-structured guides. Additional details of the study design and procedures from both stages have been described elsewhere [14–16, 20]. To ensure confidentiality, all participants were provided a participant identification number (PTID), and documents referring to names or other personal identifiers were stored separately from records identified by PTID. All documents or data transferred to the analysis team were identified by PTID only.

Questions about motivation to join the VOICE trial were asked of all participants in stage 1 and stage 2 in both the IDIs and FGDs; thus, data from both interview modalities are included in this paper. The guides included the following questions: “Can you tell me all the reasons that you joined VOICE?”, “What made you join the VOICE trial?”, and for women with low drug detection in stage 2, “What made you stay in the trial, despite not taking your products?” Additional probing topics explored how personal benefits (health care, reimbursements), personal life (partners, family, work), and/or community factors (community opinions about research or study) influenced their motivation to join the trial, and whether motivations changed as a result of their randomization group or other factors. Audio recordings of the IDIs and FGDs were transcribed into the local language, then translated into English, and checked throughout the process for quality and accuracy.

### VOICE-D: Data analysis

Demographic data was used to create a socio-economic status (SES) indicator variable, described in Table 1, to explore potential socio-economic differences by country. Analysis for country difference of study sample characteristics were done using Fisher’s Exact test for categorical variables and Kruskal-Wallis test for continuous variables. Qualitative interviews were coded by a team of analysts using NVivo10 qualitative software using guidebooks developed for each stage of analysis. A subset of the codes that were most representative of the primary research questions (11 for stage 1 and 13 for stage 2) were used to compute inter-coder reliability of ≥80% among 10% of all transcripts. Coded data were then summarized into memos by thematic area and interpretations were critically discussed to reach group consensus [14, 16].

For this secondary analysis, code reports for the “JOIN” or “INTEREST” codes from Stages 1 and 2 respectively, were reviewed and summarized by the lead author into categories of motivations to join and remain. After the initial aggregate analysis of the data, a by-country analysis was completed to examine any differences by site. Findings were entered into a matrix organized by country and motivation to join or remain and were discussed with two senior co-authors to reach consensus on interpretation. Findings were subsequently reviewed by all co-authors to confirm interpretation and gain insight from field collaborators. To further protect participant confidentiality, all names used to reference participants in this paper are pseudonyms that were assigned by the lead author during analysis. Any words or phrases in quotations indicate a direct quote from the participant.

The study protocol was approved by the Institutional Review Boards at RTI International and at each of the study sites and was overseen by the regulatory infrastructure of the U.S. National Institutes of Health and the Microbicide Trials Network (MTN).

## Results

### Participant characteristics

VOICE-D study sample characteristics (*n* = 171) are presented in Table 1 by study country. South Africans were significantly younger (median age = 25) than those from Uganda and Zimbabwe (median age = 29) and more likely to be unmarried (0% married compared to 88% in Zimbabwe and 59% in Uganda). Overall, of the participants who were unmarried, most had a primary sexual partner (86%). Ugandan participants, who included more sex workers than other sites, had significantly more lifetime sexual partners (median = 5) and they were more likely to know that their partners had other partners (56%), while South Africans and Zimbabweans were more commonly unsure (79 and 76% respectively). A higher proportion of South African participants were in the highest SES bracket (47%), while Ugandans were more likely to be in the lowest bracket (76%). Most participants said they earned their own income (73%), though less so in Zimbabwe (*n* = 54%). Overall, 91% of women said their partner provided them with financial or material support. We previously published that VOICE-D stage 2 participants were younger and less well educated, but otherwise, demographically and behaviourally similar to other VOICE participants at the same sites [14]. Also previously published were findings that the TVB/TOB respondents differed from the rest of the VOICE trial participants in that they were less likely to be from the tenofovir oral and gel arms and were disproportionately from Uganda and Zimbabwe than from South Africa [19].

### VOICE-D: Qualitative findings on motivations to participate

The dominant themes from the VOICE-D qualitative interviews were that, 1) women joined and remained in VOICE primarily to receive free personal health benefits; 2) women who stated they joined the trial for altruistic intentions also identified personal health benefits as a motivator; 3) participants’ friends were influential in motivating them to join as well as propagating misinformation on the benefits of the study products; and 4) despite not using the products consistently, a sense of fulfilling their commitment to the study through adhering to study visits and procedures kept some women in the trial. Differences by country are highlighted as they are relevant. Due to the retrospective nature of the study, reasons for joining and reasons for remaining are presented together unless specifically stated by the participant to be one or the other.

### Participating for personal health benefits

Women in VOICE-D commonly reported that they participated in VOICE to receive personal health benefits. Receiving HIV testing and preventative healthcare, at no cost to them, were the most regularly discussed benefits. Other personal benefits mentioned by many participants included HIV risk reduction counseling, treatment for illnesses, receiving the study product for HIV prevention, and, for many fewer, reimbursement.

#### Repeat HIV testing

The promise of repeat HIV testing or “knowing [one’s] status” was the most consistently stated motivation to participate in VOICE. As one participant said, *“I like having my blood taken for testing so that I will know where I stand”* (Bheka, IDI, South Africa). Many women identified the regular HIV testing as a way to continually monitor their HIV status and therefore mitigate their HIV-related worry throughout the study. In Uganda, the monthly testing (and subsequent negative results) was also described specifically as a salient reason for remaining in the trial.

*What made me stay in the research study was that each time I came, they would test my blood and give me the negative results and I also benefited from the other treatment they offered to me each time I told them the pain I had…* (Charity, FGD, Uganda).

The desire for regular HIV tests was often related to a widespread suspicion that male partners had other sexual partners and subsequent fear that they would acquire HIV from their partners.

*I realized that if I did not join VOICE, I would put my life at risk; in that I could contract a disease [HIV/AIDS] unawares because my husband is the kind of person who always has girlfriends [extra-marital affairs]. So, I concluded that if I joined VOICE I would always know my health status…I was also tested for other sexually transmitted diseases.* (Kudzai, IDI, Zimbabwe).

An additional benefit of the regular HIV testing received in the trial involved the feeling that women could confirm their partner’s fidelity or reveal his infidelity. Participants explained that she could tell her partner that if she came back with positive results from her HIV tests then his lack of fidelity would be revealed. Some women invoked this reasoning to encourage sexual behavior change from their partners, while others used this as a method to encourage their partners to come to the clinic to get tested as well.

*I came to the study so that I could be told whether I had the HIV virus. When they tested me and found that I was safe [not HIV infected] I was happy because I was healthy. I went home and told my husband that there was an association/organization [referring to clinic] and that he should also be tested. He didn’t refuse. He came and was also tested and both of us were found to be safe [HIV negative].* (Kijai, IDI, Uganda).

For some, participation in VOICE was a chance to get tested for the first time. One participant said, *“I don’t want to lie, I really gained a lot…It was also the first time I had an HIV test. I had never tested before”* (Thabisile, IDI, South Africa). Furthermore, some participants recognized that women, particularly young women, have difficulty using or getting their partners to use condoms consistently, making it all the more crucial to know their status:

*I also heard that we were going to get tested for many things. It’s easy to not use a condom and that’s what also worried me. As young people we sometimes forget to use condoms but being in the research has kept me well.* (Khule, IDI, South Africa).

#### Health care evaluations, treatment and counseling

The ability to monitor one’s overall health, by receiving comprehensive evaluations and preventative health care, was another personal health benefit many women discussed as motivating to participate in the study. For example, when asked what enticed her, Afiya explains:

*R: The fact that you get medical check-ups for your health status, ways of family planning, blood check-up, and any other information about your health.*

*I: Uhm [ok]. Did they carry out tests on you?*

*R: Yes.*

*I: What exactly where they checking for?*

*R: HIV/AIDS, Yellow/Liver fever, pregnancy tests and in case you have any sickness, they give you treatment. For example, I came when I had some pain in my fallopian tubes, but I was thoroughly treated.* (Afiya, IDI, Uganda).

The availability of preventative health checks, such as pap smears, helped women get diagnosed and treated for issues they otherwise would not have discovered.

*Yes, and she [staff] explained to me. She told me what is good about it [the research]. Sometimes you don’t get a chance to do a check-up. I didn’t even know what a pap-smear is before…But then I found out. That helped me because they found something wrong with me.* (Thabisile, IDI, South Africa).

In South Africa and Uganda predominantly, healthcare was commonly mentioned as a motivator to stay in the trial despite challenges using the products. Diana experienced side effects from the study gel and plainly explains:

*Personally, I wanted to access that treatment and that is why I remained in the trial in spite of the itching. Because I was well taken care of. The other reason is that I thought I had one of those diseases but God helped me. When I was checked, I was not found with Candida or any other disease. But I also saw those with diseases being treated. I also came so that if I was found with diseases, I would receive treatment.* (Diana, FGD, Uganda).

A few participants, more prominently in South Africa, also discussed the benefit of receiving health counseling as a motivator to join the study. This arose often in the context of the education, counseling, and linkage to care if one of the various diagnostic tests they received during the trial detected something. One woman describes how she was worried about testing positive for HIV but, nonetheless, was encouraged to test, knowing that if she were to become infected she would be linked to care and counseling through the study:

*Yes, I was encouraged. I was afraid before but by the time I joined [VOICE] I was more at ease because I had been told that if the results are not good [HIV positive], I will get counselling, they will explain things to me and I won’t have any problem.* (Bheka, IDI, South Africa).

#### Perceived preventative benefits

Desiring protection from HIV through use of the study products was highlighted by women across all sites, though not necessarily as the sole reason for wanting to join.

*I always liked to participate in research; on your arrival to participate, your status was being checked [HIV blood test] for your own knowledge. I also liked protecting myself from the virus [prevent HIV acquisition] through the study products we used.* (Lesedi, IDI, South Africa).

Remaining in the trial due to a belief that the products were effective was also reported by some women. As this participant states simply, “*The products are the reason why I stayed…They give me protection from HIV.*” When asked why she was different than the other participants who didn’t use the product, she went on to explain, “*It could be that I believe that if you use the product it will protect you, it could be that they did not have that attitude”* (Lindelani, IDI, South Africa).

Other women, however, understood and acknowledged that the products were under investigation; they relied on the regular HIV testing to reduce their HIV worry. Still, these women hoped that the study products would provide some protection. Anesu explains, when asked how worried she was about getting HIV while in VOICE:

*It was the same but the thought that you got tested, like every month…It calmed me down. But then, the fact that it was a study also. So it’s natural to wonder about whether it works or [laughing] it doesn’t. So I think it was an issue of striving and hoping, I hope it works [laughing].* (Anesu, IDI, Zimbabwe).

This potential for protection was linked to the hope for successful trial results and motivated some, especially in Zimbabwe, to remain in the trial to know its outcome.

#### Reimbursement and healthcare costs

While a few VOICE-D participants mentioned money (visit and/or transport reimbursement) as a motivator to enroll in the trial, few reported it as their sole rationale, as exemplified in the following dialogue:

*I: Were you motivated by anything you stood to gain from taking part in the research?*

*R: Money.*

*I: Money? Okay. Is there anything else?*

*R: It was money and wanting to know my status.* (Anelisa, IDI, South Africa).

In contrast, other participants, in Uganda specifically, explained how the financial reimbursement had no influence on their decision to join. In Sanyu’s experience, when she decided to join VOICE she was unaware that she would be given any transport money:

*I: Is there any other motivation for your joining the study?*

*R: No…Because when I was coming, I was not told that there was some money…If I had been told, maybe one would say that I came to make some money…No.*

*I: Didn’t your friend tell you about the money?*

*R: She just told me that it was voluntary.*

*I: Mmm.*

*R: She didn’t tell me that there was some money involved.* (Sanyu, IDI, Uganda).

While money was seldom overtly mentioned as a primary reason for joining or remaining in the trial, the expenses averted by receiving free HIV testing and health services was an important factor for many, particularly in Zimbabwe and Uganda. Although women wanted to know their HIV and general health status, diagnostic tests were not affordable outside of the trial setting, as explained below:

*Because they used to take off blood and you would know the state of your health. That is what was most attractive to me...In other places, I would have been charged for the tests and I did not have money during that time.* (Dembe, IDI, Uganda).

Another participant explains the benefit of free health check-ups:

*Let me just say I don’t earn a lot of money, so I didn’t have enough money to go for medical check-up, but I could get it [checkup] here [at the study clinic] for free.* (Namukasa, IDI, Uganda).

### Social networks influence

Social networks played an important role in recruitment into VOICE as demonstrated by the many women who explained that they joined due to encouragement from a participant with whom they were friends.

*It was actually a friend of mine that initially brought the program to my attention. I asked her what the program was all about, and she advised me that I should go there and get more information about the program. And if the opportunity arose, I should then join the program. (Uchi, IDI, Zimbabwe).*

These social networks may have also contributed to therapeutic misconception, namely dissemination of false information that the product was already proven efficacious, discounting the placebo-controlled design and investigational nature of the trial. In the example below, the participant explains that she joined because of a friend, and that friend was convinced to join by another friend because she had been promised protection, indicating a snowball effect of both recruitment and distorted information.

*She [friend] heard about it from someone else who had taken part in the research and she took me there. They accepted us at the research…She must have told her that, ‘if you take part in the research, you can be safe.’* [..] *Because they test you most of the time and give you something [product] to protect yourself with. And for real, when I was still participating in the study, I was protected and my life was all right.* (Nobuntu, IDI, South Africa).

### Joining for altruism

A handful of participants expressed that they joined VOICE to help other women and future generations, or to “save the world” by helping test the product, which we define for this analysis as an altruistic motivation. As Mbira explains:

*I found it to be good because they said they were trying to find out if there was a drug which was able to protect women from contracting the disease [HIV infection] through sex. So it’s within my rights to join, though I might not enjoy the benefits myself, it might benefit my child or another woman like me.* (Mbira, IDI, Zimbabwe).

Many participants who reported wanting to help others, however, also mentioned concomitant personal health reasons for wanting to join. This included using the product for protection, getting health care, regular HIV testing, family planning, etc. This was the case for Nofoto, who said:

*One of the reasons why I decided to join VOICE was that I could see that women could be helped to prevent becoming infected with HIV, if they use the products provided. I also got a chance to get tested and know my status. I also wanted to know more about what to use so as to prevent myself from falling pregnant.* (Nofoto, IDI, South Africa).

### Committed to the study and wanted study results

As described in the segments above, a dominant theme for remaining in VOICE was for the personal health benefits, however, a few described a sense of dedication and commitment towards VOICE itself that is worth mentioning. For example:

*Me, I think that because of the way we had made a contract with MTN [Microbicide Trial Network], it was hard to breach it. You had decided and signed that you would stay until study ends. So you had to finish the study.* (Sharon, FGD, Uganda).

These women’s sense of fulfilling their commitment to the study was centered around returning for their visits and contributing their time.

*What motivated me stay in the study despite not using the products? ... It was just wanting to get to the end of the study. […] Yes, because we had an agreement that when you enter you must, you must finish off. However, if you were disinterested; some just left in the middle of the study if they had lost interest in the study.* (Mudiwa, IDI, Zimbabwe).

Furthermore, a few participants discussed remaining in VOICE to find out about the study results stayed despite product misgivings, as Zendaya describes here:

*R: I wanted to see the results of the trial. At the end if it will be a success or not.*

*I: Alright. You wanted to see if the trial was going to be successful or not?*

*R: Ummmh. In addition, I was also gaining a lot of useful knowledge from the trial.*

*I: You were gaining knowledge? What did you struggle most with, related to taking the products?*

*R: I was worried that the product might have significant side effects and I would put my health in danger.* (Zendaya, IDI, Zimbabwe).

### VOICE: TVB/TOB quantitative findings on motivation to participate

Of the 171 VOICE-D participants, 106 completed the VOICE termination questionnaire (Table 2). The most commonly selected reason for joining the trial was “to help test a product that may prevent women from getting HIV” (95%), while HIV education, HIV testing, and free or better health care were also frequently selected (88, 84, and 69% respectively). We investigated these survey responses to corroborate the qualitative findings from VOICE-D. The VOICE-D participant responses were also compared with the responses from VOICE participants at the VOICE-D sites who were not enrolled in VOICE-D (*N* = 684), using Fisher’s exact test. Table 2 presents the data in order of frequency, which is the same for VOICE-D participants and VOICE participants at the VOICE-D sites.

**Table 2:** Responses to VOICE termination visit survey (TVB/TOB) on motivation(s) to join VOICE at VOICE-D sites

	Participated in VOICE-D at VOICE-D Sites	
	Yes (*n* = 106)	No (*n* = 684)
Motivation to join VOICE (multiple answers allowed)		
To help test a product that may prevent women from getting HIV	101 (95%)	660 (97%)
To get information about HIV prevention	92 (88%)	618 (91%)
To be tested for HIV	89 (84%)	603 (89%)
For the free or better quality health care during the trial	72 (69%)*	555 (82%)*
For the financial reimbursement	13 (13%)	73 (11%)
Other		
*Yes*	0 (0%)	2 (< 1%)
*No*	95 (100%)	640 (100%)

*Bold text signifies

*p* < 0.05 using Fisher’s exact *p* value

## Discussion

This analysis of VOICE-D women’s accounts as to why they participated in the VOICE trial, highlights the multifaceted nature of trial participation, and likely misalignment in priorities between study participants and researchers. Participants’ reflections from the qualitative interviews in VOICE-D on their original motivations to join VOICE ranged, though primarily focused on receiving free personal health benefits (e.g. HIV testing and healthcare). Data from the VOICE termination visit questionnaire provide a slightly different picture, as the most frequently reported reason for joining was altruistic (i.e. help test the product). Qualitatively, women who stated altruistic motivations often also identified personal health benefits as motivators to join VOICE. Joining for financial reimbursement was not commonly mentioned qualitatively or quantitatively. Motivations to stay in VOICE, reported qualitatively only, stemmed mostly from enjoying the continued personal health benefits, particularly healthcare, but sometimes from a feeling of commitment to the study and desire to know study results. Additionally, this analysis found that social networks were influential in recruitment to the study and in spreading inaccurate information about the efficacy of study products.

That participants joined the trial primarily for personal health benefits – such as free preventative health care and treatment, and repeat HIV testing – demonstrates a desire to stay healthy and monitor one’s HIV risk, and is consistent with prior literature from other active product microbicide trials with African women [23, 24], and other HIV prevention trials [25]. Although joining for better quality health care was the fourth most common motivator from the termination visit data, it was the dominant theme qualitatively. Of note, there was a statistically significant difference in the frequency of this motivation for VOICE-D participants and VOICE participants at VOICE-D sites, though the rank order of all motivations was the same for both groups. In the qualitative analysis with VOICE-D participants, South African women, who were younger and unmarried, were also interested in receiving HIV education and counselling; the second most common motivator from the VOICE termination visit survey. One potential explanation that incorporates both the interest in testing an HIV prevention product and receiving personal health benefits as reasons to join the trial has been referred to as *conditional altruism* [26]. *Conditional altruism* asserts that people may intend to enroll in a trial to help others but ultimately will only participate if they additionally receive some personal benefit. Alternatively, while helping test the product was the most common motivation from the VOICE termination visit survey, it was perhaps not the most important, as participants could, and did, choose additional responses. Many participants reported multiple reasons for participating in the study qualitatively as well, and previous literature confirms that participants in clinical research often articulate a variety of reasons for joining, including both the opportunity to obtain medical or personal benefit, *and* the opportunity to help others [27–31].

Participating for financial reimbursement was not commonly reported in either data set, which might reflect some participants’ reluctance to openly admit an interest in a monetary incentive. However, avoiding costs while receiving the preventive health care they desired, and needed, was a strong motivator for women who joined VOICE, particularly for those from Uganda and Zimbabwe who were of significantly lower income and were responsible for the care of more children than their South African counterparts. Thus, we cannot ignore the social and economic context in these trial communities, though local socio-cultural norms may also have played a role in motivations to join. Of note, HIV testing is not uncommon in these settings -- according to the World Health Organization, about half of adults in Africa reported receiving HIV testing services between 2010 and 2014 -- yet as much as 40% of people who were diagnosed through HIV testing services were not linked to care [32, 33]. Barriers to care include transportation costs, distance to facility, stigma, fear of disclosure, staff shortages, long waiting times and poor-quality services (particularly for adolescents) [32]. Women in this study highlighted similar barriers and noted reasons for preferring the study facilities, specifically the consistent access to testing as well as connection to treatment and services if one were to become infected. The fact that people join clinical trials to obtain higher quality health care than public clinics provide is consistent with findings from prior HIV prevention trials in African settings [23, 34–37]. Local clinics are overcrowded, may not offer full preventive healthcare such as pap smears, and may include direct and indirect financial costs to the patients, reducing a woman’s ability to successfully manage her health [38–42]. This imbalance between “standard of care” in trials and in local settings is often debated; nevertheless, it should be acknowledged that the World Medical Association’s revised Declaration of Helsinki endorsed the practice that all trial participants receive the worldwide standard of care [43, 44]. Thus, until there is massive health care reform in these settings, high quality health care offered in trials will likely continue to motivate many.

There are multiple factors that likely contributed to the misalignment of priorities between the researchers’ primary objectives to test if the products were safe and efficacious, and the participants primary concern to improve or maintain their health, which ultimately meant not using the products. As their desire for obtaining the study benefits like free HIV testing and health monitoring highlight, participants in VOICE-D clearly felt at risk of acquiring HIV. Yet receiving the regular health monitoring appear to have done enough to dispel or reduce their HIV worries and keep them in the trial. The risk of using an unfamiliar product of unknown efficacy, on the other hand, may have been too great, thus discouraging use of the products, at least consistently [14]. Previous literature emphasizes that benefits to the participant must be higher than the perceived risks for individuals to participate in trials [26]. This may help explain why overall retention in VOICE was high, but adherence was low [10]. Also, while there were no consequences of nonuse (e.g. being forced to exit the trial), participants used various means of deception to hide their product nonuse, fearing to be terminated from the study and lose their healthcare and HIV testing benefits, or fearing to fail to uphold their end of the participant-research study relationship [14, 15]. Furthermore, mentioning the signed informed consent as a reason for staying in the trial suggests that for those women, adhering to their study visits and procedures was enough to fulfill their commitment. Though not found in this analysis, prior literature speculates that receiving the financial reimbursement may have played a role in boosting participants’ feeling of commitment, while also instilling a sense of obligation to appear to use the products [19, 23]. One implication from these findings is that future studies could benefit from reminding participants of their responsibilities more explicitly, potentially in a documented list of participant rights and responsibilities, to highlight that there is a difference between agreeing to participate and complying with research requirements [45]. Additionally, part of the informed consent process could involve a discussion with participants about their personal motivations to participate and how these may be best aligned with the priorities of the research.

In this analysis, friends influenced recruitment (e.g. encouraging others or being encouraged to join), and beliefs about the products’ efficacy (e.g. therapeutic misconception). As highlighted in the primary VOICE-D paper, peers also negatively affected adherence to study products, often through spreading rumors about the product [14], a reflection of the importance of social networks throughout the trial. MTN-020/ASPIRE, a more recent microbicide HIV prevention trial evaluating the effectiveness of a monthly vaginal ring [46], placed greater emphasis on motivating women through group-based peer support, creating a sense of pride for consistent ring use. Group activities, such as providing site-level aggregate adherence feedback as compared to other sites also fostered a sense of a shared goal, and one’s individual responsibility in contributing to the goal [47]. This can be explained as altruism on a smaller scale, or to use the term in the Zulu language, *Ubuntu* [48]. In other words, social networks and peer support can be used to motivate participants to help their community. The longer-term value of finding an effective product (“macrosocial” benefit of helping society at large) could be viewed as personally advantageous in the short-term by reframing it as improving social capital (“microsocial” benefit of helping one’s immediate social network) [34, 49]. The promising protective results from ASPIRE suggest that such connections between the micro- and the macro-social study benefits may improve participants’ engagement, and hence trial outcomes. In future studies, it may also benefit to explore how social networks can be used more intentionally during recruitment and then monitored throughout the study to understand effect on adherence.

This study has several limitations, the most noteworthy for the qualitative data is that women were asked retrospectively (at least a year and a half after they joined VOICE) to reflect on why they participated in the trial. This may have led to recall bias, or a reconstruction of narrative due to having left the trial and received study results. It also made it difficult to always differentiate between reasons to join and reasons to remain. As described in other analyses, social desirability bias was likely present, which may have led women to underreport their desire to join the study for financial reimbursement, for example, in addition to other measures, such as reporting product non-use [14–16]. Women articulated various reasons for joining the study, including the desire for personal health benefits, however, these may be less socially stigmatizing to disclose than a monetary interest in participation. It is worth noting, however, that VOICE-D was designed as a separate protocol and efforts were made to reduce social desirability bias and encourage honest reporting, including using non-VOICE trial staff at the sites, and allowing visits to take place in non-trial locations [14, 15]. Another limitation is that this study sample was selected based on predetermined criteria, and thus was not intended to be representative of the full VOICE study population. Finally, administration of qualitative interviews is inherently variable, which leads to differences between interviewers in the level and amount of probing. Thus, one participant may have been asked about multiple motivations to join, while another was probed for details about one particular motivation. However, given the large sample size for this qualitative study, the range of responses, as well as the fact that the results corroborate findings from previous studies, it suggests that the limitation around probing is of limited concern. The major limitations from the termination visit survey data is that the form was administered at the end of multi-year trial about reasons for joining the trial, and thus participants may have been influenced by their experience in the trial. Additionally, the form was administered after the early stopping of two of the study products for futility [10], so we do not have data from any participants who had already exited the study. Further, implementation of the form in South Africa was delayed by the IRB process, resulting in over-representation from Uganda and Zimbabwe sites [19].

## Conclusions

This analysis contributes to the literature on motivations to participate in clinical trials by providing a more nuanced understanding of the social, contextual, and individual factors that influenced women’s reasons for participating in a phase 2b HIV prevention trial, despite low product use. Participants primarily joined VOICE for personal health benefits such as free healthcare and HIV testing, which highlighted their desire to protect their health and monitor their HIV risk. A desire to maintain these benefits, and a sense of commitment to the trial, motivated women to remain in the trial. Appealing to participants’ commitment to the study and reminding them that using the product as instructed is implicit in that commitment may increase participants adherence. Additionally, building off successes like the ASPIRE trial [46, 47], further research should be conducted to investigate how best to utilize social networks to improve adherence to study products. Ultimately, understanding the various reasons why individuals join and remain in studies will help align the priorities of both researchers and participants.

## Abbreviations

FGD: focus group discussion
IDI: in-depth interviews
MTN: Microbicide Trials Network
PrEP: Pre-Exposure Prophylaxis
TVB/TOB: Termination visit Vaginal or Oral product Behavioral questionnaire

## References

[1] van der Straten A, Van Damme L, Haberer JE, Bangsberg DR (2012). Unraveling the divergent results of pre-exposure prophylaxis trials for HIV prevention. AIDS.

[2] Masse BR, Boily MC, Dimitrov D, Desai K (2009). Efficacy dilution in randomized placebo-controlled vaginal microbicide trials. Emerg Themes Epidemiol.

[3] Karim QA, Baxter C, Karim SA (2014). Microbicides and their potential as a catalyst for multipurpose sexual and reproductive health technologies. BJOG.

[4] Celum C, Baeten JM (2012). Tenofovir-based pre-exposure prophylaxis for HIV prevention: evolving evidence. Curr Opin Infect Dis.

[5] Anderson PL, Kiser JJ, Gardner EM, Rower JE, Meditz A, Grant RM (2011). Pharmacological considerations for tenofovir and emtricitabine to prevent HIV infection. J Antimicrob Chemother.

[6] Baeten JM, Donnell D, Ndase P, Mugo NR, Campbell JD, Wangisi J, Tappero JW, Bukusi EA, Cohen CR, Katabira E (2012). Antiretroviral prophylaxis for HIV prevention in heterosexual men and women. N Engl J Med.

[7] Grant RM, Lama JR, Anderson PL, McMahan V, Liu AY, Vargas L, Goicochea P, Casapia M, Guanira-Carranza JV, Ramirez-Cardich ME (2010). Preexposure chemoprophylaxis for HIV prevention in men who have sex with men. N Engl J Med.

[8] Hendrix CW, Chen BA, Guddera V, Hoesley C, Justman J, Nakabiito C, Salata R, Soto-Torres L, Patterson K, Minnis AM (2013). MTN-001: randomized pharmacokinetic cross-over study comparing tenofovir vaginal gel and oral tablets in vaginal tissue and other compartments. PLoS One.

[9] Baeten JM, Haberer JE, Liu AY, Sista N (2013). Preexposure prophylaxis for HIV prevention: where have we been and where are we going?. JAIDS J Acquir Immune Defici Syndr.

[10] Marrazzo JM, Ramjee G, Richardson BA, Gomez K, Mgodi N, Nair G, Palanee T, Nakabiito C, van der Straten A, Noguchi L (2015). Tenofovir-based preexposure prophylaxis for HIV infection among African women. N Engl J Med.

[11] Rees H, Delany-Morelwe S, Lombard C (2015). FACTS 001 phase III trial of pericoital tenofovir 1% gel for HIV prevention in women. Conference on retroviruses and opportunistic infections (CROI): 2015.

[12] Van Damme L, Corneli A, Ahmed K, Agot K, Lombaard J, Kapiga S, Malahleha M, Owino F, Manongi R, Onyango J (2012). Preexposure prophylaxis for HIV infection among African women. N Engl J Med.

[13] Koss CA, Bacchetti P, Hillier SL, Livant E, Horng H, Mgodi N, Mirembe BG, Gomez Feliciano K, Horn S, Liu AY, et al. Differences in Cumulative Exposure and Adherence to Tenofovir in the VOICE, iPrEx OLE, and PrEP Demo Studies as Determined via Hair Concentrations. AIDS Res Hum Retroviruses. 2017;00(00):1-6. 10.1089/aid.2016.0202 PMC5564054 28253024

[14] van der Straten A, Montgomery ET, Musara P, Etima J, Naidoo S, Laborde N, Hartmann M, Levy L, Bennie T, Cheng H (2015). Disclosure of pharmacokinetic drug results to understand nonadherence. AIDS.

[15] Montgomery ET, Mensch B, Musara P, Hartmann M, Woeber K, Etima J, van der Straten A (2017). Misreporting of product adherence in the MTN-003/VOICE trial for HIV prevention in Africa: Participants' explanations for dishonesty. AIDS Behav.

[16] Musara P, Montgomery ET, Mgodi NM, Woeber K, Akello CA, Hartmann M, Cheng H, Levy L, Katz A, Grossman CI, et al. How Presentation of Drug Detection Results Changed Reports of Product Adherence in South Africa, Uganda and Zimbabwe. AIDS Behav. 2018;22(3):877-86. 10.1007/s10461-017-1685-x PMC5587392 28110473

[17] Rossi L. Microbicide Trials Network Statement on Decision to Discontinue Use of Oral Tenofovir Tablets in VOICE, a Major HIV Prevention Study in Women. https://mtnstopshiv.org/news/mtn-statement-decision-discontinue-use-oral-tenofovir-tablets-voice-major-hiv-prevention-study; 2011.

[18] Rossi L. Microbicide Trials Network Statement on Decision to Discontinue Use of Tenofovir Gel in VOICE, a Major HIV Prevention Study in Women. In. https://mtnstopshiv.org/news/mtn-statement-decision-discontinue-use-tenofovir-gel-voice-major-hiv-prevention-study-women; 2011.

[19] Mensch BS, Brown ER, Liu K, Marrazzo J, Chirenje ZM, Gomez K, Piper J, Patterson K, van der Straten A (2016). Reporting of adherence in the VOICE trial: did disclosure of product nonuse increase at the termination visit?. AIDS Behav.

[20] Duby Z, Hartmann M, Mahaka I, Montgomery ET, Colvin CJ, Mensch B, van der Straten A (2014). Language, terminology and understanding of anal sex amongst VOICE participants in Uganda, Zimbabwe and South Africa. AIDS Res Hum Retrovir.

[21] Duby Z, Hartmann M, Montgomery ET, Colvin CJ, Mensch B, van der Straten A. Condoms, Lubricants and Rectal Cleansing: Practices Associated with Heterosexual Penile-Anal Intercourse Amongst Participants in an HIV Prevention Trial in South Africa, Uganda and Zimbabwe. AIDS Behav. 2016;20(4):754-62. 10.1007/s10461-015-1120-0 PMC4698090 26126586

[22] Duby Z, Hartmann M, Montgomery ET, Colvin CJ, Mensch B, van der Straten A. Sexual scripting of heterosexual penile-anal intercourse amongst participants in an HIV prevention trial in South Africa, Uganda and Zimbabwe. Cult Health Sex. 2016;18(1):30-44. 10.1080/13691058.2015.1064165 PMC4659730 26223703

[23] Minnis AM, van der Straten A, Salee P, Hendrix CW (2016). Pre-exposure prophylaxis adherence measured by plasma drug level in MTN-001: comparison between vaginal gel and Oral tablets in two geographic regions. AIDS Behav.

[24] Wong C, Parker C, Ahmed K, Agot K, Skhosana J, Odhiambo J, Makatu S, Lemons A, Mueller M, Van Damme L et al. Participant motivation for enrolling and continuing in the FEM-PrEP HIV prevention clinical trial. In: IAS: 2013; Kuala Lumpur, Malaysia; 2013.

[25] Mensch BS, Friedland BA, Abbott SA, Katzen LL, Tun W, Kelly CA, Sarna A, Srikrishnan AK, Solomon S (2013). Characteristics of female sex workers in southern India willing and unwilling to participate in a placebo gel trial. AIDS Behav.

[26] McCann SK, Campbell MK, Entwistle VA (2010). Reasons for participating in randomised controlled trials: conditional altruism and considerations for self. Trials.

[27] Giguere R, Zimet GD, Kahn JA, Dolezal C, Leu C-S, Mabragaña M, McGowan I, Carballo-Diéguez A. The Motivations and Experiences of Young Women in a Microbicide Trial in the USA and Puerto Rico. World journal of AIDS. 2013;3(3):1-12. 10.4236/wja.2013.33023 PMC3855411 24324918

[28] Meque I, Dube K, Bierhuizen L, Zango A, Veldhuijzen N, Cumbe F, Feldblum PJ, van de Wijgert J (2014). Willingness to participate in future HIV prevention trials in Beira, Mozambique. Afr J AIDS Res.

[29] Tharawan K, Manopaiboon C, Ellertson C, Limpakarnjanarat K, Chaikummao S, Kilmarx PH, Blanchard K, Coggins C, Mastro TD, Elias C (2001). Women's willingness to participate in microbicide trials in northern Thailand. J Acquir Immune Defic Syndr (1999).

[30] Thienkrua W, Todd CS, Chaikummao S, Sukwicha W, Yafant S, Tippanont N, Varangrat A, Khlaimanee P, Holtz TH (2014). Prevalence and correlates of willingness to participate in a rectal microbicide trial among men who have sex with men in Bangkok. AIDS Care.

[31] Wendler D, Krohmal B, Emanuel EJ, Grady C, Group E (2008). Why patients continue to participate in clinical research. Arch Intern Med.

[32] Organization WH (2015). Consolidated guidelines on HIV testing services: 5Cs: consent, confidentiality, counselling, correct results and connection 2015.

[33] Organization WH (2015). Fact sheet to the WHO consolidated guidelines on HIV testing services.

[34] Dhalla S, Poole G (2014). Motivators to participation in actual HIV vaccine trials. AIDS Behav.

[35] Stadler JJ, Delany S, Mntambo M (2008). Women's perceptions and experiences of HIV prevention trials in Soweto, South Africa. Soc Sci Med (1982).

[36] Stirratt MJ, Gordon CM (2008). Adherence to biomedical HIV prevention methods: considerations drawn from HIV treatment adherence research. Curr HIV/AIDS Rep.

[37] Macphail C, Delany-Moretlwe S, Mayaud P (2012). It's not about money, it's about my health': determinants of participation and adherence among women in an HIV-HSV2 prevention trial in Johannesburg, South Africa. Patient Prefer Adherence.

[38] Akinyemiju TF, McDonald JA, Lantz PM (2015). Health care access dimensions and cervical cancer screening in South Africa: analysis of the world health survey. BMC Public Health.

[39] Chandler CIR, Kizito J, Taaka L, Nabirye C, Kayendeke M, DiLiberto D, Staedke SG (2013). Aspirations for quality health care in Uganda: how do we get there?. Hum Resour Health.

[40] Chevo T, Bhatasara S. HIV and AIDS Programmes in Zimbabwe: implications for the health system. ISRN Immunology 2012, 2012:11.

[41] Musheke M, Ntalasha H, Gari S, Mckenzie O, Bond V, Martin-Hilber A, Merten S (2013). A systematic review of qualitative findings on factors enabling and deterring uptake of HIV testing in sub-Saharan Africa. BMC Public Health.

[42] Mayosi BM, Benatar SR (2014). Health and health Care in South Africa — 20 years after Mandela. N Engl J Med.

[43] Lie RK, Emanuel E, Grady C, Wendler D (2004). The standard of care debate: the declaration of Helsinki versus the international consensus opinion. J Med Ethics.

[44] Marouf FE, Esplin BS (2015). Setting a minimum standard of Care in Clinical Trials: human rights and bioethics as complementary frameworks. Health Hum Rights.

[45] Resnik DB, Ness E (2012). Participants’ responsibilities in clinical research. J Med Ethics.

[46] Baeten JM, Palanee-Phillips T, Brown ER, Schwartz K, Soto-Torres LE, Govender V, Mgodi NM, Matovu Kiweewa F, Nair G, Mhlanga F, et al. Use of a Vaginal Ring Containing Dapivirine for HIV-1 Prevention in Women. N Engl J Med. 2016;375(22):2121-32. 10.1056/NEJMoa1506110 PMC4993693 26900902

[47] Montgomery ET, van der Straten A, Chitukuta M, Reddy K, Woeber K, Atujuna M, Bekker LG, Etima J, Nakyanzi T, Mayo AJ (2017). Acceptability and use of a dapivirine vaginal ring in a phase III trial. Aids.

[48] Amico KR, Wallace M, Bekker LG, Roux S, Atujuna M, Sebastian E, Dye BJ, Elharrar V, Grant RM (2017). Experiences with HPTN 067/ADAPT study-provided open-label PrEP among women in Cape Town: facilitators and barriers within a mutuality framework. AIDS Behav.

[49] Sivaram S, Zelaya C, Srikrishnan AK, Latkin C, Go VF, Solomon S, Celentano D (2009). Associations between social capital and HIV stigma in Chennai, India: considerations for prevention intervention design. AIDS Educ Prev.

[50] Vyas S, Kumaranayake L (2006). Constructing socio-economic status indices: how to use principal components analysis. Health Policy Plan.

[51] Luecke EH, Cheng H, Woeber K, Nakyanzi T, Mudekunye-Mahaka IC, van der Straten A, Team M-DS (2016). Stated product formulation preferences for HIV pre-exposure prophylaxis among women in the VOICE-D (MTN-003D) study. J Int AIDS Soc.

